# Unexpected Genetic Cause in Two Female Siblings with High Myopia and Reduced Visual Acuity

**DOI:** 10.1155/2018/1048317

**Published:** 2018-05-23

**Authors:** M. N. Preising, C. Friedburg, W. Bowl, B. Lorenz

**Affiliations:** Department of Ophthalmology, Justus-Liebig University Giessen, University Medical Center Giessen and Marburg GmbH, Giessen Location, Germany

## Abstract

In daily life, myopia is a frequent cause of reduced visual acuity (VA) due to missing or incomplete optical correction. While the genetic cause of high myopia itself is not well understood, a significant number of cases are secondary to hereditary malfunctions or degenerations of the retina. The mechanism by which this occurs remains yet unclear. Two female siblings, 4 y and 2 y, respectively, from a consanguineous Pakistani family were referred to our department for reduced VA and strabismus. Both girls were highly myopic and hence were further examined using standard clinical tests and electroretinography (ERG). The latter confirmed confounded electrical coupling of photoreceptors and bipolar cells. Further inquiry and testing confirmed a similar condition for the father including impaired night vision, reduced VA, photophobia, and an equally characteristic ERG. Findings in the mother were unremarkable. Subsequent genetic analysis of autosomal recessive and X-linked genes for congenital stationary night blindness (CSNB) revealed a novel homozygous splice site mutation in* CACNA1F* in the two girls transmitted from both the father and the mother. While in males the above clinical constellation is a frequent finding, this report, to the authors' knowledge, is the first demonstrating biallelic mutations at the* CACNA1F* locus in females.

## 1. Introduction

Reduced visual acuity (VA) occurs in several conditions, ranging from retinal degeneration [1, 2], insufficient cone system function [3, 4], and morphological underdevelopment of the fovea [5] to unfocussed image projection onto the fovea [6]. Of the latter, a simple but frequent cause is unbalanced longitudinal growth of the eye leading to high myopia [6].

Whereas myopia in the sense of axial growth in itself can lead to macular degeneration, patients with high myopia should always be investigated for nystagmus, strabismus, and night vision impairment, since these may be signs of dysfunctions or degeneration of the cone and rod system.

If in the disease course the dysfunction leads to degeneration, milder disease imposes as rod-cone or cone-rod degeneration (RCD, CRD) whereas very early or even infancy onset with severely reduced ERG responses in the first year of life is called Leber congenital amaurosis (LCA) or early-onset severe retinal dystrophy (EOSRD) based on the extent of visual dysfunction. These patients may be myopic, too.

On the other end of the spectrum, there is stationary disease. Congenital stationary night blindness (CSNB) is a heterogeneous group of retinal dysfunction caused by autosomal traits (13 recessively inherited genes, 3 dominantly inherited genes) and X-linked traits (*NYX* and* CACNA1F*) [7]. Based on the extent of rod system dysfunction in the Ganzfeld ERG, CSNB was categorized into the rare Riggs-type with rod photoreceptor dysfunction, and the much more frequent Schubert-Bornschein-type with a complete (CSNB1) and an incomplete form (CSNB2). Complete CSNB1 results from mutations in* NYX* and affects ON-bipolar function with impaired generation of the amplitude of the scotopic *b*-wave while incomplete CSNB2 affects impulse transmission from photoreceptors to bipolar cells due to mutations in* CACNA1F* and severely reduces *b*-wave amplitudes [7]. Hemizygous males present with a broad spectrum of visual dysfunction [8].

Here we report on two Pakistani girls who were referred for severely reduced visual acuity, nystagmus, and high myopia. ERGs were suggestive of, and the genetic analysis revealed, a* CACNA1F *mutation in the homozygous state. To the best of our knowledge this has never been reported, and the extent of dysfunction in the presence of homozygous mutations in* CACNA1F *is remarkable.

## 2. Materials and Methods

Best corrected visual acuity (BCVA) was evaluated using age adapted tests (Lea symbols, Cardiff crowding cards, Teller acuity cards, Landolt rings) after testing for and correcting refractive errors (Nidek Autorefractor, Nidek Technologies, Padova, Italy).

Fundus images were taken with a Zeiss FF450 camera (Carl Zeiss Meditec, Jena, Deutschland) and fundus autofluorescence (FAF) at 488 nm was recorded on a Spectralis HRA (Heidelberg Engineering, Heidelberg, Germany).

Retinal stratification was assessed by Spectral-domain Optical Coherence Tomography (SD-OCT, Spectralis HRA Heidelberg Engineering, Heidelberg, Germany), as well as by a hand-held SD-OCT (Envisu, BioptigenTM, Leitz, Wetzlar, Germany). SD-OCT data was quantitatively analyzed using the DIOCTA software [11]. Whenever possible volume scans were recorded and analyzed but in the young patients age related capacity and nystagmus allowed assessment by single scan recording only. Also, ideal positioning of the single scans over the fovea was not generally possible.

Ganzfeld electroretinograms (ERG) were recorded using DTL-electrodes according to ISCEV standards [12] on an Espion Unit (Diagnosys LLC Cambridge, UK).

Visual fields were tested employing a Goldmann perimeter (GVF, Haag-Streit Switzerland, Bern, Switzerland) and the MP1-Microperimeter (Nidek Technologies, Padova, Italy). MP1-microperimetry was performed as previously reported [9] with 200 ms stimuli of Goldmann size III position within an 8° visual field (Figures 4(c)–4(f)). Goldmann visual fields were digitalized by Image J [13] and plotted with Sigma Plot 10.0 (Systat Software Inc, San Jose, CA, USA).

The index case was genotyped at Bioscientia GmbH Ingelheim, Germany, for variations in autosomal and X-linked genes underlying CSNB using a next generation sequencing (NGS) panel approach [14]. Segregation in the family was confirmed by Sanger sequencing.

Informed consent according to the tenets of the declaration of Helsinki was taken from the parents. The study was approved by the Ethical Review Board of the Medical Faculty of the Justus-Liebig-University Giessen (149/07).

## 3. Results

**Figure 1:**
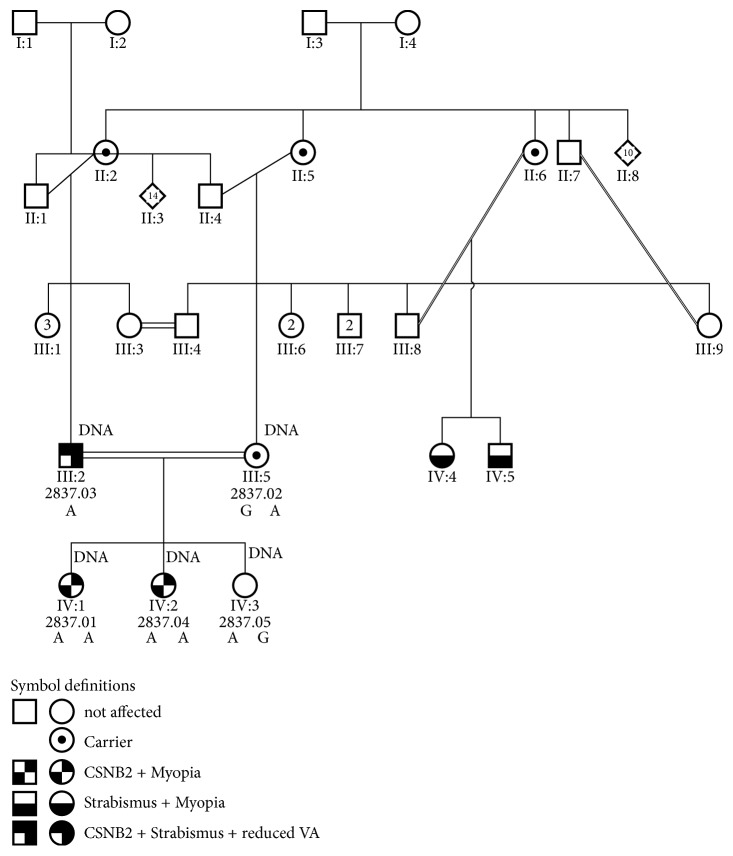
*Pedigree of family 2837*. The index case and all tested members of the family are labelled (DNA). The pedigree includes several consanguinity loops giving rise to two further affected individuals in the branch of the mother. Double line: consanguineous marriage. Further symbol definitions as given in the figure. Diamond = unknown sex, circle = female, square = male, open symbols = unaffected, number in symbols = number of individuals.

**Figure 2:**
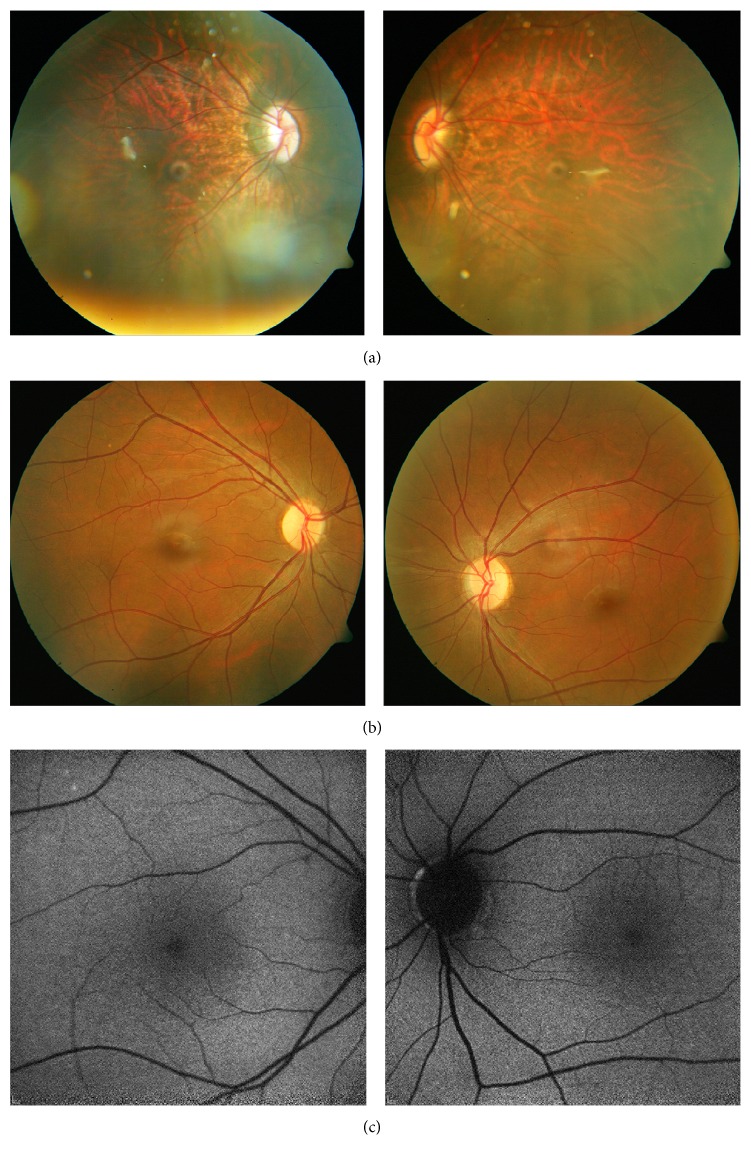
*Fundus appearance of the index case 2837.01 and her father 2837.03*. (a) Index case at age 4.25 y: stretched posterior pole including oblique entry of the optic nerve, typical for high axial length myopia. Refraction measurements in mydriasis at several visits ranged from −16 dpt to −12.5 dpt spherical equivalent. (b) Her father (at 26.75 y): slightly pale optic nerve head. The periphery was unremarkable. (c) Fundus auto fluorescence in the father was unremarkable.

A 4.25 y old daughter of a consanguineous Pakistani couple (Figure 1, 2837.01) was referred for further examinations for exophoria and nystagmus with reduced BCVA. The mother mentioned a squint in the father, too. Night problems or photophobia were denied upon specific request. This first examination revealed high myopia with BCVA in both eyes of 0.12 (Table 1). The posterior pole appeared myopic, and pigmentation around the fovea was approximately even. The overall pigmentation was within the normal intense range of Pakistani individuals (Table 1; Figure 2(a) with unchanged aspect at age 5). Recording the FAF was severely impeded by the nystagmus and provided no useful data. SD-OCTs demonstrated an almost unremarkable stratification but a shallow fovea and reduced overall thickness.

**Figure 3:**
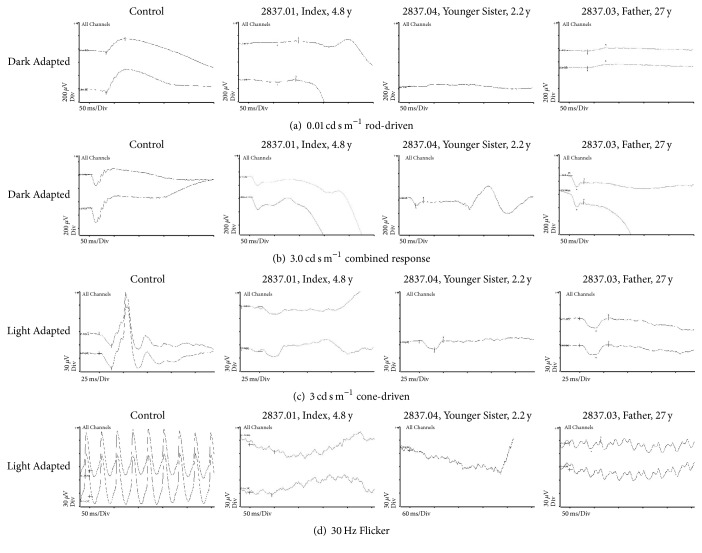
*Ganzfeld Electroretinography (ERG)*. Four standard test conditions, two in the dark and two with rod suppression, were employed, as given (photopic flash intensities; OPs merely indistinguishable, omitted), in control subjects and the three patients. The response of the rod system was severely but not totally reduced. (a) The, mostly bipolar, response in the dark is severely reduced in all three patients. (b) The *a*-wave is affected less than the *b*-wave. Hence, the latter remains below the isoelectric line (“negative response”). (c) The *a*-wave under photopic conditions is longer because positive components that give rise to a steep *b*-wave in the control subjects are smaller and initiate later. The subsequent negative deflection is missing. (d) The flicker response in all patients was below 20% of normal and clearly double-peaked in the father. The father had severely reduced rod and bipolar responses at age 26,75 y. Cone responses to single flashes and 30 Hz flicker were clearly reduced in amplitude, and the latter had a double-peak waveform.

Because of the nystagmus and high myopia, an ERG was scheduled to exclude a retinal dystrophy (Figure 3): The rod-driven response, especially from the bipolar cells, was severely reduced compared to healthy controls (Figures 3(a) and 3(b): *a*-wave to ~50%, *b*-wave ~10% of norm) indicating impeded transmission. In addition photopic responses were severely reduced, diminishing the *b*- more than the *a*-wave.

**Figure 4:**
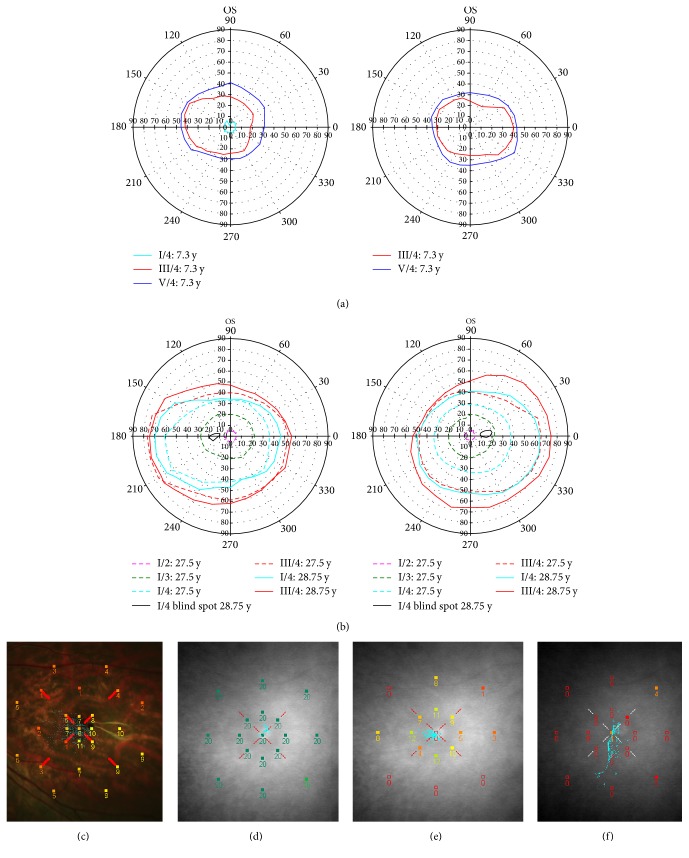
*Visual field in the index case and her father*. (a) Goldmann perimetry in index patient 2837.01 could be obtained mostly from fixation saccades and was concentrically reduced for all targets (I/4, III/4 ca. 20 to 40° radius, and V/4). (b) Her father had only slightly reduced isopters, even in reevaluation a year later (Figure 4(b), max. 73° temporal extend for III/4). (c) MP1 with white Goldmann III target from index patient 2837.01: her sensitivity in mesopic conditions was decreased by 17 dB (foveal normal value 27 dB). (d) MP1 as in (c) but from father 2837.03: his sensitivity was higher than the operating range of the MP1 (20 dB, “ceiling effect”). Method detailed in [9]. (e) Same as (d) but with a dimmer, red Goldmann III stimulus: central sensitivity reached 4 dB which is 11 dB below normal. No central relative scotoma was present. Method detailed in [9]. (f) MP1 from father with a dimmer (scotopic) background: Background and stimulus were blue and detection hence favoured rods. The stimulus was detected only just at those 5 positions marked with filled squares. Control patients yielded 11 dB, i.e., they are about 11 dB more sensitive in the dark [10].

**Figure 5:**
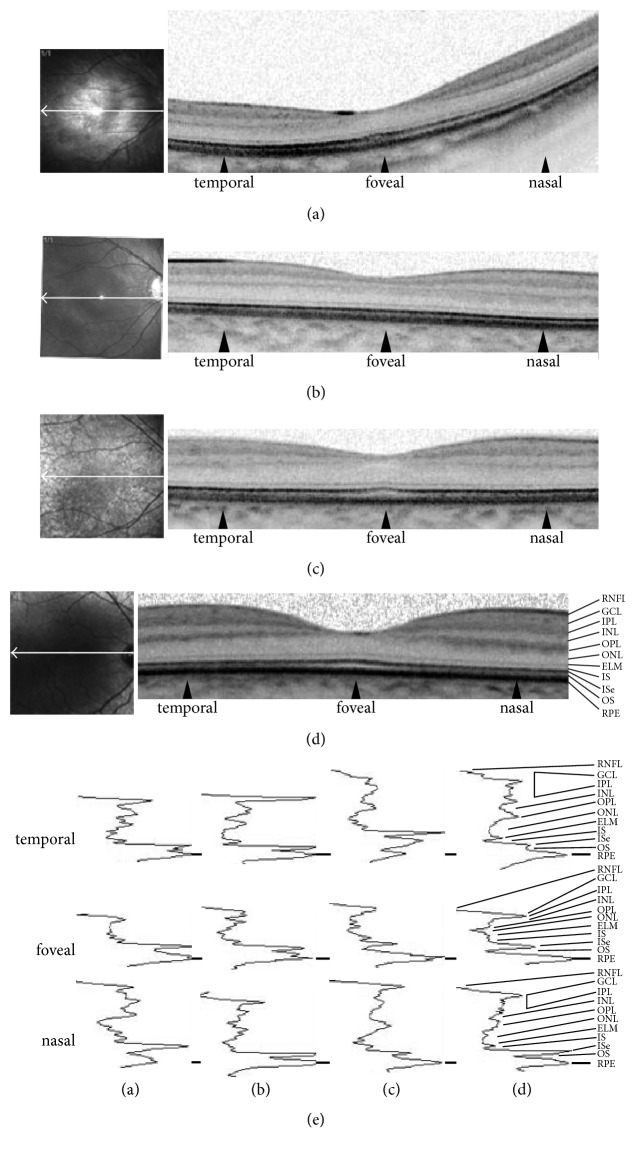
*Optical Coherence Tomography (OCT): A-scans and B-scans*. (a–d) Single scan OCT recorded on a Spectralis HRA (Heidelberg Engineering, Heidelberg, Germany) from the right eyes of the homozygous index case ((a) 2873.01) at age 7.3 y, her homozygous sister ((b) 2837.04) aged 4.2 y, the hemizygous father ((c) 2837.03) aged 28.8 y, and an unaffected control patient aged 6.5 y (d). The infrared fundus image at the left illustrate scan positions of the corresponding OCT-scan. In panel (b) the scan is slightly below the fovea and hence outer plexiform and ganglion cell layer are slightly thicker (see lower panel (b) and Figure 6). All retinal layers can be clearly distinguished. (e) For a detailed analysis DIOCTA software [11] was used to extract a-scans at the fovea and flanking positions 200 scans to each side marked with black arrow-heads in (a–d) (see corresponding labelling below). A-scans are aligned to each other at the RPE peak. Presence of all retinal layers is supported by this panel but reduction of retinal thickness is obvious for the homozygous females in the temporal A-scans and less pronounced in the foveal and nasal A-scans. RNFL: retinal nerve fiber layer, GCL: ganglion cell layer, IPL: inner plexiform layer, INL: inner nuclear layer, OPL: outer plexiform layer, ONL: outer nuclear layer, ELM: external limiting membrane, IS: inner segment layer, ISe: inner segment ellipsoid zone, OS: outer segment layer, and RPE: retinal pigment epithelium.

**Figure 6:**
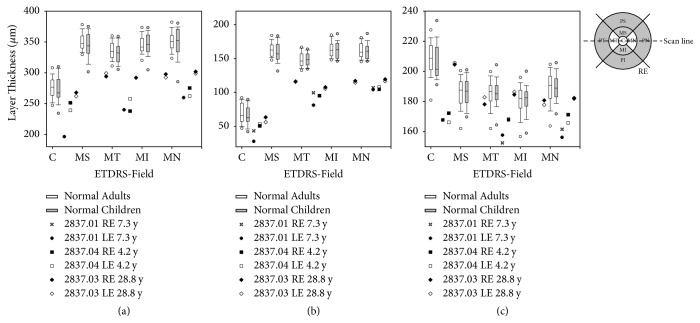
*Optical Coherence Tomography (OCT): quantitative analysis of retinal stratification*. DIOCTA [11] analysis of retinal stratification and layer thickness from the patients' OCT-scans in Figure 5 and from the corresponding scans of the left eye of the two daughters (2837.03 and 2837.04; symbols as provided). (a) Whole retina, (b) inner retinal layers (RNFL to INL), and (c) outer retinal layers (OPL – RPE) compared with boxplots of data from controls. The ordinate represents the thickness of the retinal layer measured as mean thickness of each scan in a given ETDRS field (see ETDRS grid for localization). Single scans cover values for three fields only (C: foveal, central; MT: macular circle, temporal; MN: macular circle, nasal) while volume scans additionally cover the macular, superior, and macular, inferior field (MS and MI). Peripheral fields (see ETDRS grid for comparison) were not investigated. Box plots display distribution of layer thickness values measured from volume scans of control children between 3 y and 18 y of age and control adults above the age of 18 y. Boxes enclose the 25th to 75th percentiles, whiskers the 10th to 90th percentiles and the horizontal line in each box marks the median. Limited cooperation in patient 2837.04 caused off-foveal positioning of the single scan line and hence the inner retinal layers thickness is increased. Compared to their father, thickness values from the two homozygous daughters for all retinal layers in the central field were mostly below the 25th percentile and less of normal control children. Except for the central retinal field, thickness was clearly reduced in all three patients, for the whole retinal thickness, but more pronounced for the inner retinal layers ((b) and (c)).

High myopia remained stable within the 3 years up to the latest examination at age 7.3 y while her BCVA improved probably due to her personal development (Table 1). Retinal sensitivity was strongly reduced: In Goldmann visual fields (Figure 4(a)) loss of overall sensitivity was apparent as a concentric constriction to about 20° to 40° deg radius for the Goldmann III target. Macular sensitivity was reduced by 17 dB as measured with the MP1 (Figure 4(c)). SD-OCT images remained unchanged (Figure 5(a)). Due to her good cooperation some limitations due to her nystagmus could be overcome, and an almost perfect foveal position of the single scans was obtained. DIOCTA automated analysis of foveal and parafoveal ETDRS fields (in temporal and nasal direction) revealed a general reduction of the overall retinal thickness below the 10th percentile of normal arising from all layers with an emphasis on the inner retinal layers (RFNL to OPL) (Figure 6).

In summary, clinical data in the index patient demonstrated severe loss of visual acuity and retinal sensitivity and together with the ERG pointed towards incomplete CSNB. Genetic testing was initiated and identified a homozygous splice donor variation (c.3825+1G>A, Refseq: NM_005183.3) in the X-linked* CACNA1F* gene. The variant disrupts the splice donor site of exon 31 causing translation into intron 31 terminating in a stop codon in-frame after 69 novel codons. The predicted product is truncated by 35% removing the C-terminus including the second half of the fourth transmembrane domain. The variant has not been published previously nor is it listed in variant databases. Since the variant causes a preterm stop codon nonsense mediated decay (NMD) is to be predicted as well as a major structural impact on any gene product that escapes NMD thus supporting severe functional impairment. On the other side* CACNA1F* function is fine-tuned by an extensive number of splice variations especially at intron 32 [15]. In this regard the splice site mutation at intron 31 identified in this study may allow for a gene product that does not fully abolish channel activity but retains functional channels even though at minor activity.

Segregation was confirmative with the homozygous state in her sister, and the hemizygous state in the father (typical male patient for X-linked incomplete CSNB). The mother was identified as a heterozygous carrier for the same mutation as a result of her consanguinity with her husband (Figure 1).

The younger sister first presented at the age of 2.2 y, i.e., after the genetic diagnosis in the older sister had already been confirmed. Her BCVA, refraction (Table 1), and hand-held SD-OCT recordings (data not shown) were comparable to her elder sister and were confirmed with later SD-OCT recordings at age 4.2 y (Figure 5(b)). Detailed measurements of whole retinal thickness placed her in the lower range of normal distribution with the limitation that the single scans were positioned parafoveal due to her limited cooperation which resulted in a higher thickness for the central field (Figure 6).

The ERG had the same characteristics as in the sister supporting the diagnosis of incomplete CSNB (Figure 3, 2837.04). Nystagmus and strabismus were not present (Table 1).

Gathering all information of the father after genetic confirmation, his attendance to our strabological clinic at the age of 21 came to our attention, five years before the initial visit of his daughter, for intermittent exotropia and reduced BCVA to 0.5 (Table 1). At that time, he had been slight myopic with significant astigmatism, he had had no nystagmus, and on specific request he had noted only mild photophobia and minor night vision problems that could not be further quantified. The peripapillary retinal nerve fiber layer (RNFL) thickness had been locally reduced to lower mid-normal values but this was not obvious without detailed retinal layer analysis. Squint surgery was suggested, but at that time the patient did not wish further investigations or procedures and was lost for follow-up.

The work-up examinations initiated at age 27, after the diagnosis in his daughter was made, did not reveal major changes in these data. FAF and fundus photography were unremarkable (Figures 2(b) and 2(c)). Visual fields were slightly constricted to about 70 deg temporal for Goldmann III/4 (Figure 4(b)) and sensitivity reduced by about 11 dB in mesopic as well as scotopic conditions (Figures 4(e) and 4(f)).

Retinal stratification and reflectivity in SD-OCT appeared unremarkable (Figure 5(c)). Quantitative DIOCTA analysis of the SD-OCT, however, revealed a reduction in overall retinal thickness resulting mostly from the inner retinal layers. The changes were similar to that in his daughters but less pronounced (Figure 6).

Finally, his ERG, too, was consistent with the diagnosis of incomplete CSNB but the amplitudes were larger than in his daughters (Figure 3, 2837.03).

The mother and the youngest sister aged 1.5 (Figure 1) were heterozygous carriers and all ophthalmological examinations were unremarkable.

## 4. Discussion

To the authors' best knowledge, this is the first report of a homozygous pathogenic variation in* CACNA1F *in females. The functional differences between the hemizygous father and the girls are quite apparent even though the effect of the mutation should result in comparable expression of CACNA1F. Both affected daughters (homozygous) were highly myopic and had severely reduced BCVAs. The father (hemizygous) in contrast had medium reduced BCVA with marginal myopia. He hardly appeared impaired by this or night vision disturbance or sensitivity loss in daylight conditions. Nevertheless, judged from ERG amplitudes and thresholds obtained with the MP1, the functional disturbance was significant. This highlights the importance of functional clinical investigation of seemingly unaffected family members even after careful history taking.

SD-OCT is a valuable tool to obtain additional information on the retinal structure especially in CSNB. Hand-held devices are of great value in infants and toddlers allowing high-resolution imaging without the need of sedation and very good compliance as infrared light is used not inducing light aversion [16] but nystagmus and age related capacity may impede image quality.

DIOCTA analysis [11] provided the important finding that retinal thickness was reduced in all patients, though to a lesser extent in the father. A possible interpretation regarding these changes mainly involving the inner retinal layers is that the impaired transmission from the photoreceptors to the bipolar cells impedes the development of the inner retinal layers during retinal maturation. This interpretation is supported by morphological investigations by Michalakis et al. [17] in female mice with heterozygous mutations in* CACNA1F*. In these, affected and unaffected retinal vertical columns were lying side-by-side and showed significant changes of how the photoreceptors connect to second order neurons (termed “synaptopathy” in [17]). This resulted in thinning of the OPL, especially.

The intriguing remaining question is whether the hemizygous, apparently milder condition in the hemizygous father represents a lighter expression of the phenotype compared with the homozygous condition in the daughters. Unfortunately, the spectrum of visual impairment in hemizygous males itself is broad [8], and the reason for this is unknown. Chen et al. found a similar reduction in OCT-measurements of layer thickness in hemizygous males [18]. In addition, since we found no other data on homozygous females, the range of variation in homozygous females is yet to be determined.

*CACNA1F* variations have additionally been identified in large families diagnosed with Åland Island Eye Disease (AED) or X-linked Cone-Rod Dystrophy type 3 (CORDX3) [19, 20]. The phenotype of the male patients from these families does not differ considerably from the phenotype reported here and is exemplified by severely reduced visual acuity and progressive visual field loss interpreted as photoreceptor dystrophy [21–24]. Fundus appearance, retinal stratification, and retinal function are, as given here, generally stable and fundus hypopigmentation seen in AED may result from myopia as reported in this study. The underlying variations identified in AED are mostly missense mutations [24, 25] even though a large deletion covering exon 30 and flanking intronic sequence was identified in the original AED family [19].

CORDX3 resulting from* CACNA1F* variations have been reported only rarely and with individually different genetic variations and effects on the gene [20, 23]. Therefore, identification of the underlying gene and variation is necessary to interpret the clinical data in each patient with an indicative phenotype.

CACNA1F is a regulatory subunit of the Ca_v_1.4 chloride channel. It presents with several splice isoforms modulating the Ca^2+^-flow through the channel [15]. Even though no splice form covering intron 31 has been reported to date several splice isoforms of intron 32 are known including one using intronic sequence to produce a modified C-terminal part of the gene product. This allows individual expression of residual activity in each patient which can also be seen in the large families reported for AED and CORDX3.

The challenge in diagnosing young children with significant visual impairment is the multitude of conditions ranging from early-onset severe retinal degeneration [1, 2] to albinism [26] and achromatopsia [3, 4]. Also in CSNB severe visual impairment has been reported [8]. It is hence important to look out for the preservation of retinal morphology which is very high and more homogeneous in CSNB, although in patients with biallelic* GUCY2D* mutations retinal morphology may also be preserved for a long period [27]. A good but in a limited number of cases deceivable tool is layer by layer analysis of retinal SD-OCT that helps in distinguishing patients with CSNB from patients with severe degenerative diseases of the retina, from achromatopsia, and from albinism. The ERG remains that important diagnostic tool providing characteristic responses to scotopic and photopic stimuli in of the various forms of CSNB and to exclude degenerative retinal conditions.

## Figures and Tables

**Table 1:** Summary of longitudinal ophthalmological data in the Index case, her affected sister, and their father.

Patient, sex Age at examination	Visual Acuity	Refraction	Fundus photography	Fundus Auto fluorescence	SD-OCT	Ganzfeld ERG	Night Vision, Nystagmus Photophobia Strabismus	Visual field
2837.01, f 4.25 y	0.12/0.12	RE −12.0/−2.25/167 LE −11.25/−2.5/6					Ny, SExp	
4.7 y	0.2/0.12	RE −14.75/−2.5/157 LE −12.25/−2.5/5	Figure 2(a)	np		scot. Aa=, Ab↓↓, a/b↓↓ phot. Aa↓, Ab↓↓, b/a↓, Figure 3 30 Hz: ↓↓	Ny, SExp	
5.4 y	0.2/0.4	RE −11.5/−2.25/174 LE −11.0/−3.0/10		np			Ny	GVF: concentric reduction (data not shown)
7.25 y	0.16/0.2	RE −12.0/−2.0/165 LE −11.5/−2.5/180	myopic fundus	np	Figure 5(a)			GVF: concentric reduction, Figure 4(a)

2837.04, f 2.2 y	TAC 0.03/0.1	RE −10.75/−2.75/161 LE −8.75/−2.75/10				scot. A↓↓, phot. A↓↓, b/a↓↓, inner retinal changes, Figure 3 30 Hz: ↓↓		
4.2 y	LEA 0.25/0.32	RE −6.75/−3.25/161 LE −5.75/−2.25/180	myopic fundus	np	Figure 5(b)			

2837.03, m 20.9 y	0.5/0.5	RE −1.0/−2.25/172 LE −0.25/−1.75/164					NB, P, SExt	
26.75 y	0.5/0.5	RE −0.75/−2.5/177 LE 0/−2.0/173	Figure 2(b)	Figure 2(c)	Figure 5(c)	scot. Aa=, Ab↓ phot. Aa=, Ab↓↓, b/a↓, 30 Hz: ↓ Figure 3	NB, P, SExt	
27.5 y	0.5/0.4	RE −1.0/−3.0/179 LE −0.5/−2.0/170						GVF: III/4: slight temporal constriction, Figure 4(b)
28.75 y	0.53/0.5	RE −0.75/−2.5/175 LE 0.0/−1.5/165						MP1: scotopic ↓↓, Figure 4(f) Photopic: III/4: unremarkable, Figure 4(d), I/4: ↓↓, Figure 4(e)

A: amplitude, Aa: amplitude a-wave, Ab: amplitude b-wave, b/a: ratio b-wave/a-wave, 30 Hz: 30 Hertz flicker response, GVF: Goldmann visual field, LEA: LEA Symbols®, MP1: fundus controlled microperimetry, NB: night blindness, Ny: Nystagmus, P: Photophobia, phot.: photopic, scot.: scotopic, SExp: Strabismus: Exophoria, SExt: Strabismus: Exotropia, TAC: Teller Acuity Cards, ↓: reduced, ↓↓: severely reduced, ↓↓↓: below threshold, *≕* borderline, f: female, m: male, np: not possible.
